# Systematic Analysis of Differentially Expressed Maize *ZmbZIP* Genes between Drought and Rewatering Transcriptome Reveals bZIP Family Members Involved in Abiotic Stress Responses

**DOI:** 10.3390/ijms20174103

**Published:** 2019-08-22

**Authors:** Liru Cao, Xiaomin Lu, Pengyu Zhang, Guorui Wang, Li Wei, Tongchao Wang

**Affiliations:** 1 National Key Laboratory of Wheat and Maize Crop Science, College of Agronomy, Henan Agricultural University, Zhengzhou 450002, China; 2 Grain Crops Research Institute, Henan Academy of Agricultural Sciences, Zhengzhou 450002, China; 3 National Engineering Research Centre for Wheat, Zhengzhou 450002, China

**Keywords:** maize, basic leucine zipper, transcriptome analysis, duplication, abiotic stress, subcellular localization

## Abstract

The basic leucine zipper (*bZIP*) family of transcription factors (TFs) regulate diverse phenomena during plant growth and development and are involved in stress responses and hormone signaling. However, only a few *bZIPs* have been functionally characterized. In this paper, 54 maize *bZIP* genes were screened from previously published drought and rewatering transcriptomes. These genes were divided into nine groups in a phylogenetic analysis, supported by motif and intron/exon analyses. The 54 genes were unevenly distributed on 10 chromosomes and contained 18 segmental duplications, suggesting that segmental duplication events have contributed to the expansion of the maize *bZIP* family. Spatio-temporal expression analyses showed that *bZIP* genes are widely expressed during maize development. We identified 10 core *ZmbZIPs* involved in protein transport, transcriptional regulation, and cellular metabolism by principal component analysis, gene co-expression network analysis, and Gene Ontology enrichment analysis. In addition, 15 potential stress-responsive ZmbZIPs were identified by expression analyses. Localization analyses showed that *ZmbZIP17*, *-33*, *-42*, and *-45* are nuclear proteins. These results provide the basis for future functional genomic studies on *bZIP* TFs in maize and identify candidate genes with potential applications in breeding/genetic engineering for increased stress resistance. These data represent a high-quality molecular resource for selecting resistant breeding materials.

## 1. Introduction

Transcription factors (TFs) function in highly conserved network hubs and directly regulate the expression of genes to maintain a suitable living environment inside and outside the plant and control plant growth and development [1]. As one of the first steps in regulating gene expression, TFs are closely related to the proteome, the metabolome, and phenotypic groups, and studies on their functions are essential for elucidating the entire gene regulatory network of plants [2]. When plants are subjected to low temperature, drought, salt stress, or exogenous hormones, TFs are induced to bind to corresponding cis-elements through a series of signal transduction steps to activate or inhibit the generation of the RNA polymerase transcription complex. In this way, TFs regulate the expression of stress-responsive genes to mediate stress responses and improve the stress resistance of plants [3].

Basic leucine zipper (*bZIP*) TFs are a highly conserved family. Members of the *bZIP* TF family have been identified or predicted in the genomes of many eukaryotes including yeasts, animals, and plants [1,4,5,6]. In many plant genomes, members of the *bZIP* TF family are classified into subgroups according to sequence similarity, conserved motifs, and DNA binding sites. For example, the *Arabidopsis bZIP* TFs have been classified into 10 groups based on the sequence similarity of basic regions and certain conserved motifs [4]. Similarly, the *bZIP* TFs of rice have been classified into 11 groups (I–XI) on the basis of their amino acid sequences and DNA binding sites in the *bZIP* basic domain [5]. The 64 *bZIP* TFs in cucumber form six groups (I–VI) on the basis of their phylogenetic relationships [7]. The 114 *bZIP* TFs in apple have been classified into 10 subgroups, A–I and S, with the S subgroup being the largest [8]. The *Fragaria vesca bZIP* TFs have been classified into 11 subgroups: A–I, S, and U [9].

The *bZIP* TFs recognize cis-acting elements with core sequences that include the ACGT palindrome, such as TACGTA (A box), GACGTC (C box), and CACGTG (G box). Most genes induced by light or abscisic acid (ABA) contain these elements in their promoter regions [10]. In *Arabidopsis*, members of groups C and S, such as group C member AtbZIP10 and group S member AtbZIP53, produce a variety of transcriptional complexes with different regulatory characteristics in the nucleus by homo- or hetero-dimerization. A *bZIP* dimer specifically binds to the promoter gene, encoding proline dehydrogenase to activate its expression under hypotonic stress [11]. In *Arabidopsis*, other *bZIP* TFs, such as those containing transcription factor HY5 and GBF groups, specifically bind to G-box elements to activate the expression of light-induced genes [12]. Other members of the *bZIP* TF family play major roles in abiotic and biotic stress responses and seed development, primarily through the ABA signal transduction pathway [4,13,14,15]. Although many *bZIP* TFs have been identified or predicted from different species, only a few have been functionally characterized.

The *bZIP* TFs are involved in various biological processes under normal conditions. For example, they play important roles in organ and tissue differentiation [16], cell elongation [17], and somatic embryogenesis [18]. They also play important roles as regulators in responses to various biotic/abiotic stresses and signaling, such as hormone and sugar signaling [19,20,21,22], light responses [23], and osmotic stress [24]. There is increasing interest in the biological functions of *bZIP* TFs in the responses to drought, low temperature, and salt stresses. Such studies have mainly focused on *Arabidopsis* [25,26,27,28,29], rice [30,31,32,33,34,35], wheat [36], soybean [37,38], and tomato [39].

Although *bZIP* TFs are known to be involved in various stress responses, little is known about the genome-wide expression patterns of this gene family. A number of molecules that interact with TFs have been reported. While these interacting molecules can provide information about the mechanism of action of TFs, they cannot explain the evolutionary history of this gene family in maize. It would be of great significance to understand the diverse roles of multiple *bZIP* family members because of their potentially important functions in many biological processes. 

As an important cereal crop, maize has become a model plant for research on genetics, evolution, and other biological research [40]. In this paper, 54 *bZIP* genes related to drought were screened from a previously published drought-rewatering transcriptome dataset. We analyzed their phylogenetic relationships, gene structure, and chromosomal localization. We conducted gene duplication, principal component, gene co-expression network, Gene Ontology enrichment, and spatio-temporal expression analyses. These analyses identified 15 maize *bZIP* TFs that may be involved in responses to various abiotic stresses. We monitored changes in the transcript levels of these genes in response to ABA, polyethylene glycol (PEG, simulated drought), high temperature, and NaCl treatments. The results of this study enhance our understanding of the evolutionary history and functional mechanisms of members of the maize *bZIP* family. These data also represent a high-quality molecular resource for selecting and generating stress-resistant breeding materials.

## 2. Results

### 2.1. Genome-Wide Identification and Classification of bZIP Genes in Maize

We screened the drought-rewatering transcriptome using Pfam and SMART (Simple modular architecture research tool)and identified 54 differentially expressed genes with *bZIP* domains (designated as *ZmbZIP1* to *ZmbZIP54*). To investigate the structure of these genes, we used multiple online tools to identify their chromosomal location and sequence characteristics. The 54 *bZIP* genes were distributed on 10 chromosomes of maize and encoded polypeptides with 106 to 654 amino acids. The details of all predicted ZmbZIP proteins are listed in Table 1, including the chromosome location of their encoding gene, protein length, molecular weight, theoretical pI, instability index, grand average of hydropathicity (GRAVY), and gene name. The predicted molecular weights of ZmbZIP proteins ranged from 11.89 (*ZmbZIP17*) to 69.66 (*ZmbZIP9*) kDa, and the pIs ranged from 4.77 (*ZmbZIP30*) to 11.53 (*ZmbZIP49*). The GRAVY ranged from −1.171 to −0.303, indicating that these proteins are hydrophilic.

**Figure 1:**
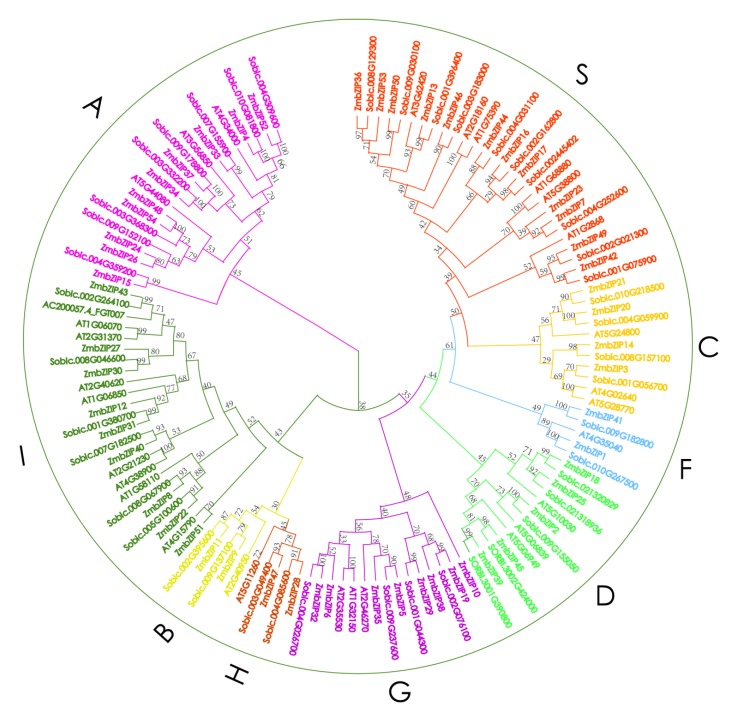
Complex phylogenetic tree of *bZIP* genes in *Arabidopsis*, sorghum, and maize. An unrooted tree is generated with the MEGA5.2 software using the amino acid sequences of the *bZIP* proteins by the neighbor-joining (NJ) method, with 1000 bootstrap replicates. The tree shows nine major phylogenetic groups (group A to S), indicated with different colored backgrounds.

To survey the extent of species-specific expansion of *bZIP* genes in maize, *Arabidopsis* (a dicot model plant), and sorghum (a model C4 monocot), we performed a joint phylogenetic analysis of their bZIP proteins. In *Arabidopsis* and sorghum, a total of 10 groups of *bZIP* TFs (A–I and S) have been classified previously [41]. The joint phylogenetic tree, including three species, grouped all *bZIPs* into nine distinct clades (A, B, C, D, F, G, H, I, and S) (Figure 1), with none in the E group. Group S had 12 members and was the largest clade, representing the 22.2% of the total ZmbZIP proteins. Groups B, F, and H had two members each. Almost all subfamilies of *bZIP* genes contained orthologs and paralogs, and subfamilies with more members had more pairs of orthologs and paralogs. Interspecific classification analyses showed that the *bZIP* genes in the three plants had undergone parallel evolution, similar to the orthologous bZIP proteins [42]. An interspecies comparison of *maize*, *Arabidopsis*, and sorghum revealed the species-specific and non-specific bZIP proteins.

### 2.2. Structure of bZIP Genes in Maize

**Figure 2:**
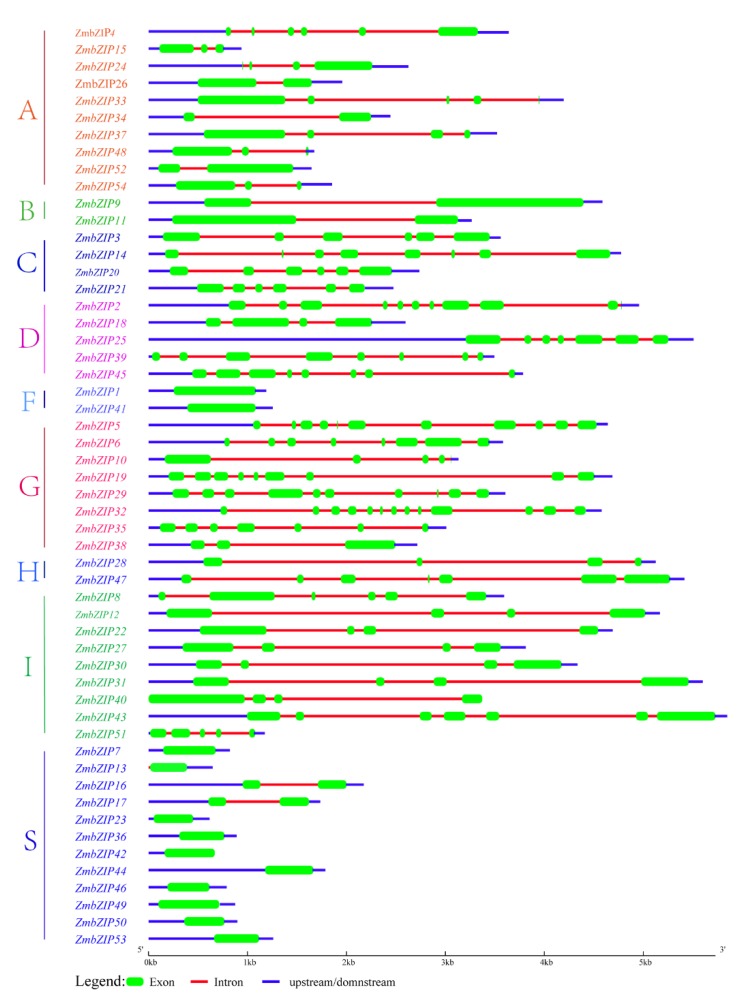
Gene structure of maize *bZIP* genes. Exon/intron organization of maize *bZIP* genes. Green boxes indicate exons and red lines represent introns, and the upstream and downstream regions are indicated by blue boxes. The sizes of exons and introns can be estimated using the scale at the bottom.

The intron/exon organization and the types and number of introns are typical imprints of gene evolution within some gene families [43]. To gain insight into the structures of the 54 maize *bZIP* genes, their exon/intron organization was investigated (Figure 2). Among the *bZIP* genes with introns, the number of introns within the open reading frame (ORF) ranged from 1 to 12. There was more variation in the number of introns in groups D (3–10 introns) and G (2–12 introns) than in other groups (generally 1–3 introns). We also found that most *ZmbZIP* genes in the same group shared highly similar exon/intron distribution patterns, including the exon length and intron numbers. For example, two genes in group B had one intron. The number of exons were similar within group S (except *ZmbZIP16* and *ZmbZIP17*) and group F. A total of 11 (20.4%) of the *ZmbZIP* genes had no intron, and all these genes were in groups S (9) and F (2). This has also been detected for *bZIP* genes in *Arabidopsis* and sorghum [41], suggesting a degree of evolutionary conservation. In summary, the exon/intron structures of *ZmbZIP* genes were generally consistent with their phylogenetic relationships.

### 2.3. Additional Conserved Motifs in bZIP Genes in Maize

**Figure 3:**
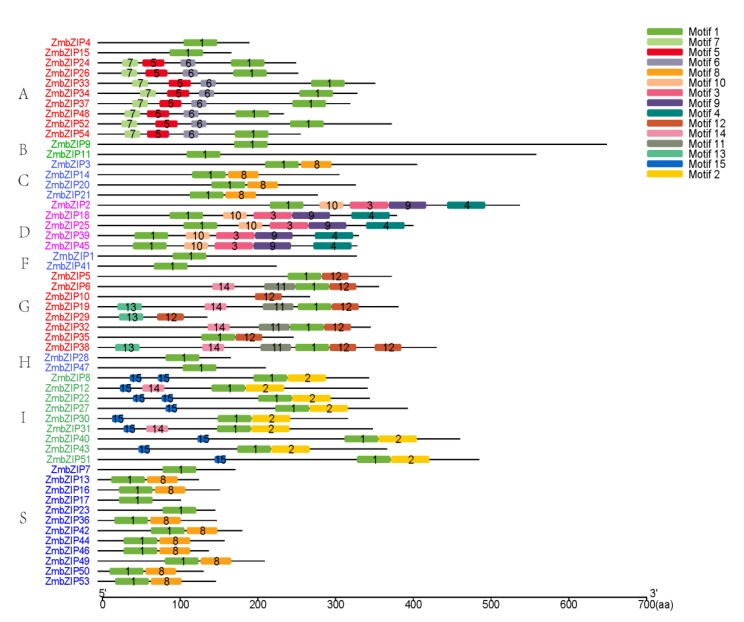
Distribution of conserved motifs in maize *bZIP* members. All motifs were identified by Multiple Expectation maximization for Motif Elicitation (MEME), using the complete amino acid sequences of *ZmbZIP* proteins. Different motifs are indicated by different color boxes numbered 1–15, and the length of each box in the proteins does not represent the actual motif size. The annotation of each motif is listed on the right.

Besides the *bZIP* domain, other conserved motifs have been detected in *bZIPs* of *Arabidopsis* [4] and rice [5]. Therefore, the 54 ZmbZIP protein sequences were analyzed using the Multiple Expectation maximization for Motif Elicitation (MEME) tool, which revealed 15 additional conserved motifs apart from the *bZIP* domain (Figure 3). Motif 1 was present in *bZIPs* in all groups, and its position was essentially the same within each group. Motif 1 was identified as a basic conserved domain shared by the *bZIP* family. Motif 8 was only detected in the C and S subgroups, indicating functional similarity between these two groups that were on the same branch in the composite evolutionary tree (Figure 1). Other motifs were specifically distributed in different groups: Motifs 5, 6, and 7 in group A; motifs 3, 4, 9, and 10 in group D; motifs 11, 12, and 13 in group G, and motifs 2 and 15 in group I. Most conserved motifs were restricted to specific groups, indicating differences among groups. Group-specific motifs can be useful for determining the specific function of each group. We also found different motifs within the same group. For example, *ZmbZIP4* and *ZmbZIP15* in group A lacked motifs 5, 6, and 7. This phenomenon was also observed in other groups, and it was suggested that there are different mechanisms of action within each group. The results of the conserved motif analysis were generally consistent with the phylogenetic relationships and classification of *ZmbZIP* genes. 

### 2.4. Chromosomal Locations and Duplications of ZmbZIPs

**Figure 4:**
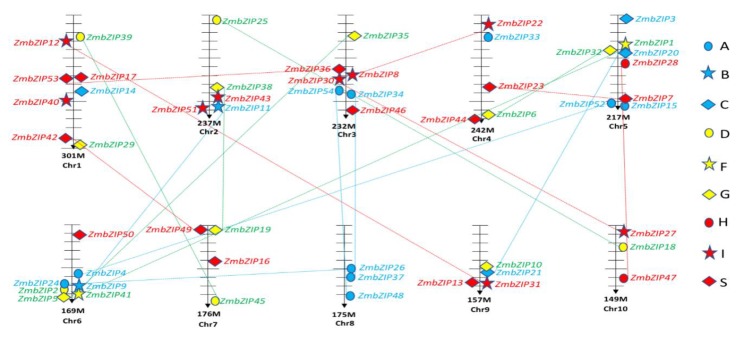
Chromosomal locations of maize *bZIP* genes. Chromosome numbers and the physical position (M) are shown at the bottom of each vertical gray bar. The segmental duplication gene pairs are joined by corresponding color lines. Different *ZmbZIP* groups are represented by different shapes and colors.

To understand how the *bZIP* gene family has grown during evolution, we analyzed the contribution of segmental repeats to the expansion of this gene family. We physically mapped all *ZmbZIPs* to the maize chromosomes and found that they were distributed among all 10 chromosomes, but not uniformly (Figure 4). Certain chromosomes and chromosomal regions had a relatively high density of *ZmbZIPs*. For example, eight *ZmbZIPs* were located on chromosomes 1 and 5, and three *ZmbZIPs* were located on each of chromosomes 8 and 10. A genome-wide analysis revealed that 36 *ZmbZIPs* were located in duplicated segments, accounting for approximately 66.7% of the total *bZIP* genes (36/54). Among the 18 segmental duplication pairs, a large proportion were located on chromosomes 5 and 6, which contained 6 and 5 segmental duplications, respectively. These results suggested that segmental genome duplication events are the main gene duplication events that have occurred in the maize *bZIP* family and have made the largest contribution to its expansion.

### 2.5. Collinearity Among Maize, Sorghum, and Arabidopsis

**Figure 5:**
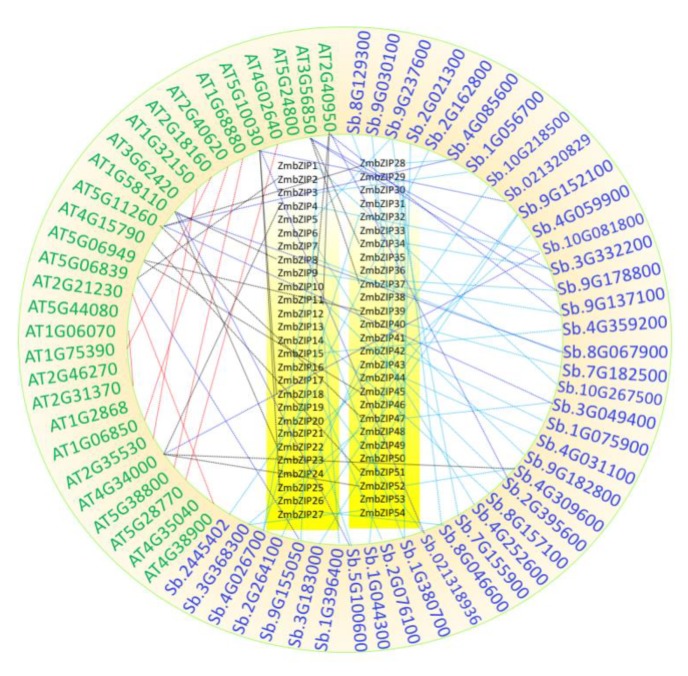
Collinearity relationships of *bZIP* genes among maize, sorghum, and *Arabidopsis*. Locus names from maize, sorghum, and *Arabidopsis* are marked with black, blue, and green fonts, respectively. Black, light blue, and dark blue lines indicate the orthologous pairs between maize and *Arabidopsis*, sorghum and maize, as well as sorghum and *Arabidopsis*, respectively. In addition, the paralogous *Arabidopsis* uses red lines.

Collinearity analyses were conducted for maize, sorghum, and *Arabidopsis* to clarify the amplification of the *bZIP* gene family in monocots and dicots. First, we analyzed the collinearity between maize and *Arabidopsis*. Nine *Arabidopsis bZIP* genes had 16 orthologs in the maize genome (black lines in Figure 5). A similar result was obtained when we aligned the *Arabidopsis bZIP* genes to the sorghum genome (dark blue lines in Figure 5). Therefore, large-scale expansion probably did not occur before the monocot-dicot split. Next, we analyzed the collinearity between sorghum and maize. In total, 46 maize *bZIP* genes had 40 orthologs in sorghum (light blue lines in Figure 5), suggesting that large-scale expansion occurred before the maize-sorghum split. This result was also reflected in the composite tree (Figure 1). These results indicated that the main amplification of this gene family did not occur before the divergence between monocots and dicots. We found seven pairs of paralogs in *Arabidopsis*, accounting for 50% of its *bZIP* genes (red lines in Figure 5); these genes are probably paralogous gene pairs that have played important roles in the amplification of the *bZIP* gene family during evolution. 

### 2.6. Cis-Element Analysis of bZIP Gene Promoter Sequences

Genes encoding TFs contain many cis-acting elements in their promoter regions that bind to other proteins and activate various pathways, for example, the ABA response signal transduction pathway and abiotic stress response pathway [44]. Therefore, identifying the cis-acting elements in the promoters of *bZIP* genes can shed light on gene function. We searched the PLACE database to investigate potential cis-acting elements in the promoter regions of the 54 *bZIP* genes in maize. In addition to some basic core components, there were multiple cis-elements in the promoter regions, such as an ABA-responsive element (ABRE), an antioxidant-responsive element (ARE), a heat-responsive element (STRE), a dehydration-responsive element (DRE), and a myeloblastosis TF binding site (MBS) (Supplemental Figure S1). These cis-elements are key components for stress responsiveness. The cis-elements of the genes differed within and among subgroups. Together, these results indicated that *bZIP* gene expression is tightly regulated in response to abiotic stress and phytohormones.

### 2.7. GO Analysis of ZmbZIP Genes

**Figure 6:**
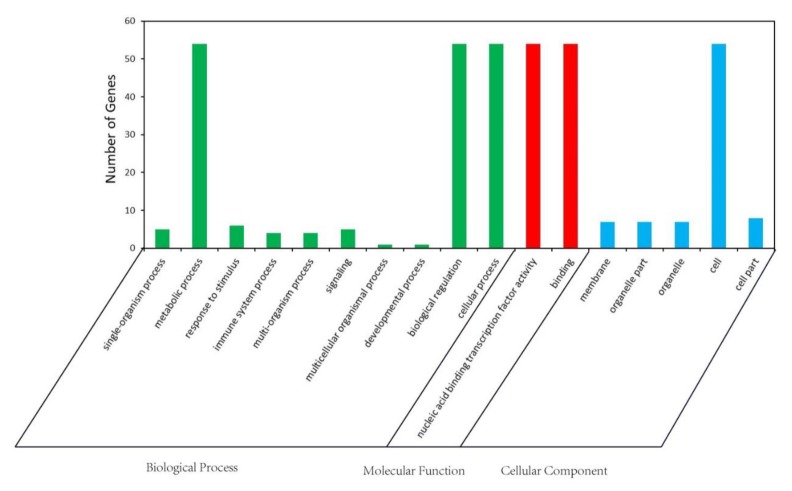
Gene ontology (GO) enrichment analysis of maize *bZIP* genes. The green, red, and blue columns represent the Biological Process, Molecular Function, and Cellular Component, respectively.

A gene ontology (GO) analysis was conducted to predict the functions of proteins encoded by *ZmbZIP* genes. The gene products were grouped into subcategories within the biological process, molecular function, and cellular component categories (Figure 6 and Table S1). In the biological process category, the significantly enriched subcategories were the metabolic process (GO: 0044710), biological regulation (GO: 0065007), and cellular process (GO: 0050794). In the molecular function category, the significantly enriched subcategories were the nucleic acid binding transcription factor activity (GO: 0001071) and binding (GO: 0005488). In the cellular component category, the significantly enriched subcategory was the cell (GO: 0044464). Group D *ZmbZIPs* were predicted to participate in the following biological processes: The single-organism process (GO: 0044699), the response to stimulus (GO: 0050896), the immune system process (GO: 0002376), the multi-organism process (GO:0051704), and the multicellular organismal process (GO: 0051239). These findings were consistent with previous reports that group D *bZIP* TFs play important roles in antioxidant and pathogen defense [4]. 

### 2.8. Expression Patterns of Maize bZIP Genes in Different Organs and in Response to Abiotic Stresses

To understand the temporal and spatial transcription patterns of *ZmbZIPs* during the maize life cycle, hierarchical clustering was performed to visualize the global transcription profile of *ZmbZIP* genes. Different maize tissues/organs and developmental stages were selected for microarray analysis, including the root, shoot, mature leaf, pollen, tassel, and embryo (Supplemental Figure S2). The heatmap showed that most of the *ZmbZIP* genes were involved in plant growth and development of maize, and their expression levels differed among organs and developmental stages. The expression patterns of some genes in the same group were different, indicating that their mechanism of action may be different.

**Figure 7:**
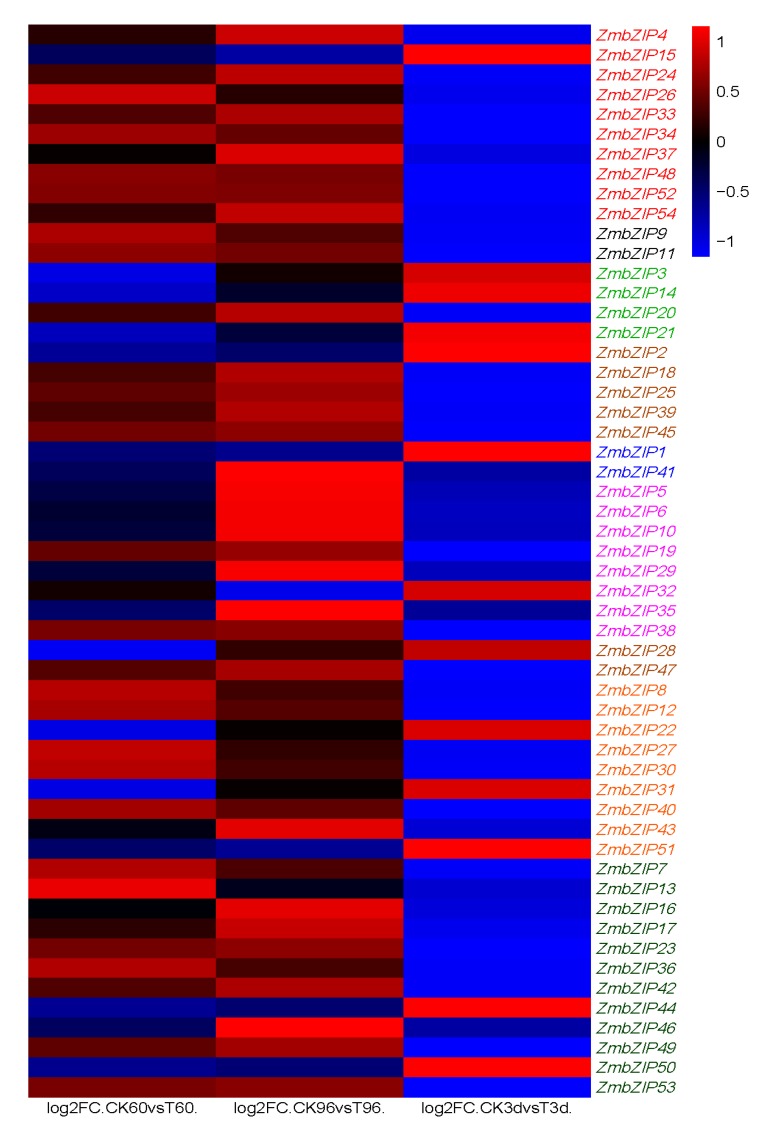
The expression pattern of 54 differentially expressed *ZmbZIPs* screened by the drought and rewatering transcriptomes. The leaves of the three-leaf stage were stressed for 60 h and 96 h by polyethylene glycol (PEG), and rewatering for 3 d denoted as T60, T96, and T3d, and the control group was named CK60, CK96, and CK3d, respectively. The FC in log2FC.CK60 vs T60 is a fold change, which is the ratio of the expression between the CK60 sample and T60. It is log2FC after taking the base 2 logarithm; the same as log2FC.CK96 vs T96 and log2FC.CK3d vs T3d. Clustering was according to groups. Different colors represent different groups (from top to bottom are groups A, B, C, D, F, G, H, I, S). Genes highly or weakly expressed in the tissues are shown in red and blue, respectively.

Comparisons of the drought and rewatering transcriptomes revealed 54 differentially expressed (|log^2^ (fold change)| ≥ 2 and false discovery rate (FDR) <0.05) *ZmbZIPs* related to drought stress. As shown in Figure 7, these genes showed opposite expression patterns between drought stress and rewatering. In other words, they were up-regulated under drought stress and down-regulated after rewatering, or vice versa. For example, group C and groups A, B, D, and G showed opposite expression trends during drought and rewatering. Further analysis found some genes within groups showed different expression patterns. For example, most members of group A showed increased expression under drought stress, and then their expression levels decreased sharply after rewatering. However, *ZmbZIP15* showed the opposite expression pattern. Similar results were detected in the other groups (groups C, D, F and G). These phenomena are consistent with the phylogenetic tree and motif analyses.

These results indicated that groups have particular functions, but the mechanism of the action can differ within each group. It is likely that individual *bZIP* genes or groups of *bZIP* genes co-operate to achieve specific functions. That is, the response of plants to abiotic stresses usually requires cross-response regulation of multi-component signaling pathways.

### 2.9. PCA and Co-Expression Network Map Analysis of ZmbZIP Genes

**Figure 8:**
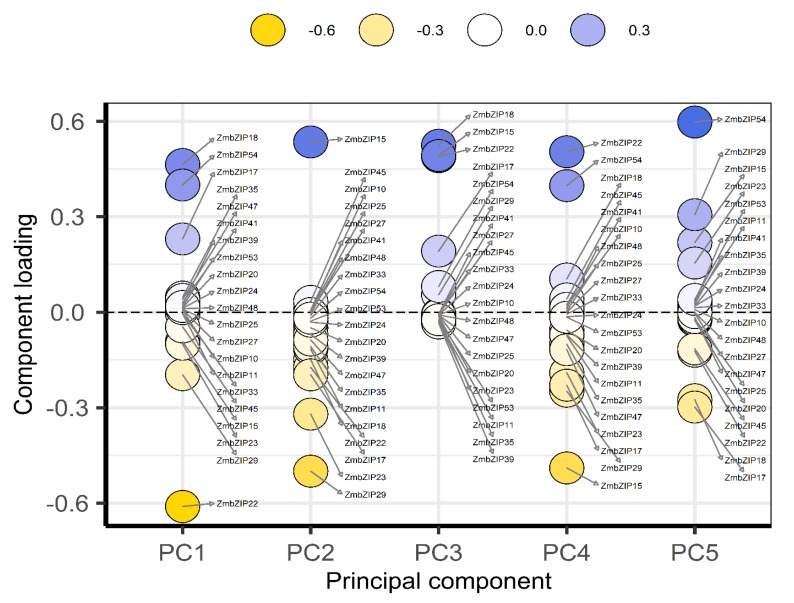
The principal component analysis (PCA) of these 54 *ZmbZIP* genes. The Pearson values of these 54 genes were used for principal component analysis (PCA). The Vertical axis represents the loadings and the horizontal axis represents the divided PCA component. The greater the absolute value of the loadings, the greater the proportion of genes in the principal component.

Principal component analysis (PCA) is a statistical procedure that converts hundreds of thousands of correlated variables (gene expression) into a set of values of linearly uncorrelated variables known as principal components. Correlation coefficients between genes were calculated based on the expression levels (Fragments Per Kilobase Exon model per Million mapped fragments (FPKM)) of 54 *ZmbZIP* genes in 18 sample materials (Supplement Table S2), and then a PCA was conducted using the internal steps of the *R* package version 3.5.3 (http://www.Bioconductor.org. We reduced the composite variable to five components, PC1 to PC5, which accounted for 42.74%, 28.69%, 11.95%, 6.85%, and 3.34% of the variation in gene expression, respectively (Supplemental Figure S3). As shown in Figure 8, in the main principal component PC1, *ZmbZIP22* and *ZmbZIP18*, had higher absolute loadings, which means that *ZmbZIP22* (negative correlation) and *ZmbZIP18* (positive correlation) is highly correlated with PC1. As shown in Figure 7, *ZmbZIP22* was down-regulated after drought stress and up-regulated after rewatering, while *ZmbZIP18* was up-regulated after drought stress, but down-regulated after rewatering. Combined with the contrary significant correlation between *ZmbZIP22* and *ZmbZIP18* in PC1, PC1 was most likely to be related to drought. We also found that the absolute values of *ZmbZIP54*, *-17,* and *-29* in PC1 are also large, and the expression patterns of *ZmbZIP54* and *ZmbZIP17* are consistent with the *ZmbZIP18*, and the expression patterns of *ZmbZIP29* and *ZmbZIP22* are identical. In other words, these genes, *ZmbZIP22*, *-18*, *-54*, *-29*, *-17*, may have higher relationship with drought and deserve further study.

**Figure 9:**
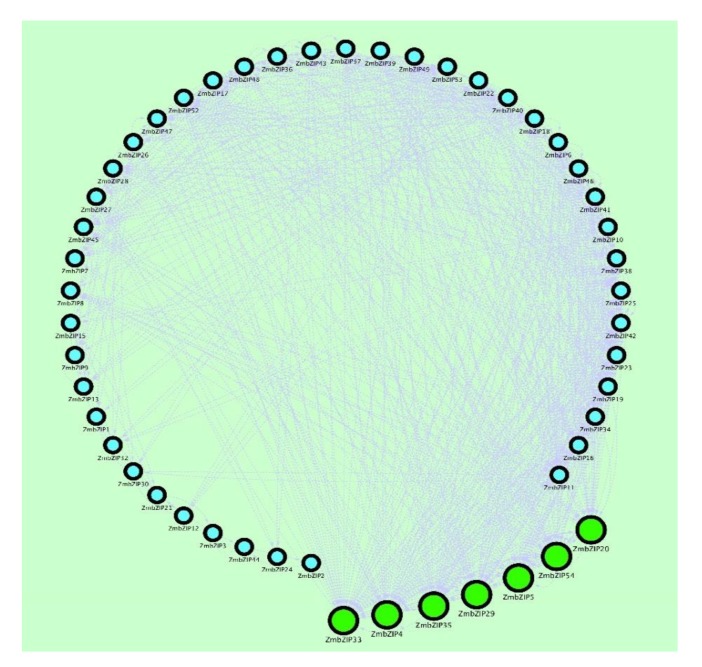
Co-expression network map of *ZmbZIP* genes. The Cytoscape software was used to map the co-expression network between 54 *ZmbZIP* genes (*p* > 0.5). The straight line represents the regulatory relationship of gene existence, and the number of relationships between a gene and surrounding genes in the network indicates that there are more genes that interact with it. A large green circle indicates that the gene is more connected to surrounding genes.

Molecular biological networks reveal interactions between molecules, which can provide details about the relationship between gene expression and regulation. In this study, genes with a correlation coefficient greater than 0.5 were imported into Cytoscape version3.7.1 (https://cytoscape.org) software to construct the gene co-expression network map. Gene co-expression network analysis produces a network diagram based on the similarity of expression among genes. The nodes in the figure represent genes, and genes with similar expression profiles connect to form a network. In this way, the possible interactions among gene products can be analyzed to understand inter-gene interactions and identify core genes. A core gene is an important hub that plays a key role in the network module. As shown in Figure 9, *ZmbZIP33*, *-4*, *-35*, *-5*, *-54*, *-29*, and *-20* were more connected with the surrounding genes, indicating that they interact with many genes. These results showed that these genes are at the core of the network and are key genes.

Both PCA and co-expression network map analyses showed that *ZmbZIP54* and *ZmbZIP29* are critical genes in maize. However, it is important to note that the results can differ depending on the focus of the analysis. The selection of these genes, combined with the results of the biological GO enrichment analysis, indicated that these are key genes involved in protein transport, transcriptional regulation, and cell metabolism. The genes identified as key genes under drought and rewatering have potential for breeding drought-resistant maize lines.

### 2.10. Expression of ZmbZIP Genes under Abiotic Stress

The *bZIP* TFs are known to regulate the expression of a wide range of stress-related genes. Figure 7 shows the heatmap of gene expression under drought and rewatering based on log2 ratio values (|log2 (fold change)| ≥ 2 of each *ZmbZIP* gene compared with the control). A recent study reported that three *bZIP* genes, *AREB1/ABF2*, *AREB2/ABF4*, and *ABF3/DPBF5* were induced by ABA, drought, and salinity in *Arabidopsis* vegetative tissues [44]. 

**Figure 10:**
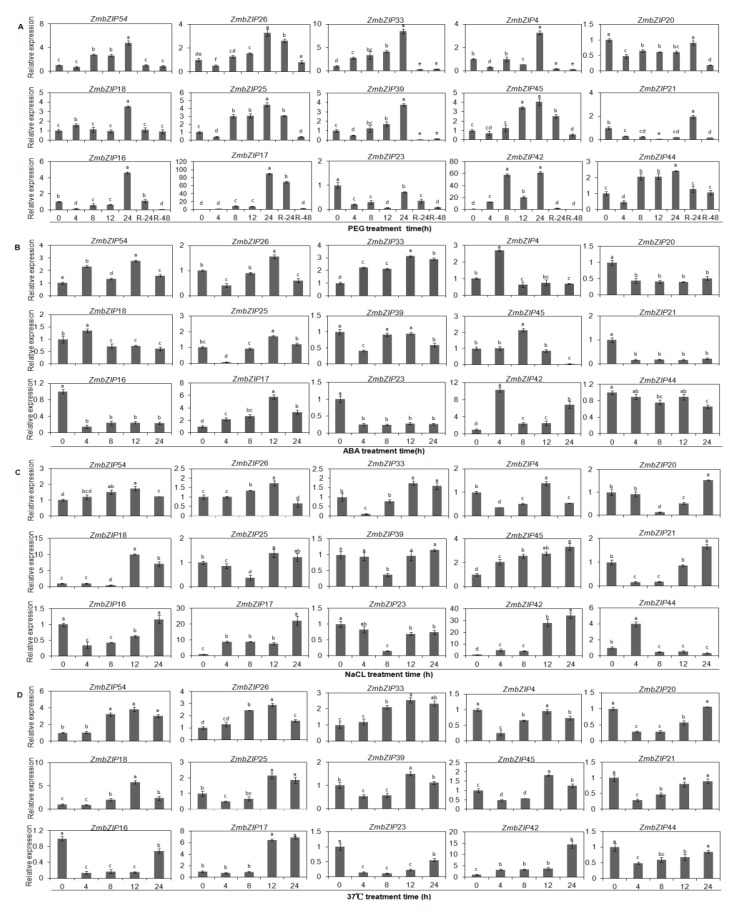
Expression patterns of *ZmbZIP* genes in response to drought, NaCl, ABA, and 37 °C treatments. The relative expression level of 15 *ZmbZIP* genes was examined by the qRT-PCR. (**A**) Relative expression of 15 *ZmbZIP* genes under PEG treatment at 0 h, 4 h, 8 h, 12 h, 24 h and rewatering at 24 h and 48 h; (**B**) Relative expression of 15 *ZmbZIP* genes under NaCl treatment at 0 h, 4 h, 8 h, 12 h, 24 h; (**C**) Relative expression of 15 *ZmbZIP* genes under abscisic acid (ABA) treatment at 0 h, 4 h, 8 h, 12 h, 24 h; and (**D**) Relative expression of 15 *ZmbZIP* genes under 37 °C treatment at 0 h, 4 h, 8 h, 12 h, 24 h. The error bars represent standard deviations (SD), *y*-axes are scales of relative expression level, and *x*-axes are the time course of treatments for each condition. Different lowercase letters indicate significant differences at *p* < 0.05 (Duncan’s test).

Through cis-element, PCA, and gene co-expression analyses, we identified maize *bZIP* genes that are likely related to drought resistance. To explore whether these genes respond to other abiotic stresses, their expression patterns were analyzed based on total RNA isolated from maize seedlings subjected to PEG, ABA, NaCl, and high temperature treatments. As shown in Figure 10, 15 *bZIP* genes (from groups A, C, D and S) were expressed at different times during the four stress treatments. The transcript levels of genes in group A (*ZmbZIP4*, *-26*, *-33*, *-54*), group D (*ZmbZIP18*, *-25*, *-39*, *-ZmbZIP45*), and group S (*ZmbZIP16*, *-17*, *-42*, *ZmbZIP44*) tended to increase during the PEG treatment, peaking at 24 h. However, at 1 d and 2 d after rewatering, their transcript levels dropped sharply. Members of group C (*ZmbZIP20*, *-21*) were down-regulated under stress, but their transcript levels peaked at 1 d after rewatering. 

The transcript levels of *ZmbZIP26*, *-33*, *-54*, *-25*, and *-17* increased during the ABA treatment, and peaked at 12 h. The maximum transcript levels of *ZmbZIP4*, *-18*, and *-42* were at 4 h of the ABA treatment. The transcript levels of *ZmbZIP20*, *-21*, *-16*, and *-23* were down-regulated during the ABA treatment. 

All the *bZIP* genes except for *ZmbZIP23* tended to show increased transcript levels under salt stress, with peak expression at different times. Under heat stress (37 °C), the transcript levels of *ZmbZIP26*, *-17*, *-18*, *-25*, *-45*, *-54*, *-33*, and *-42* increased to peak levels 3- to 14-times higher than that in the control.

Comparisons of the relative expression levels of these genes under four abiotic stress treatments showed that some genes in the same group had different response patterns, and different groups of genes could show similar response patterns. These analyses also showed that *ZmbZIP17*, *-33*, *-42*, and *-45* were strongly induced under all four stress treatments.

### 2.11. Subcellular Localization of ZmbZIPs

The genes *ZmbZIP17*, *-33*, *-42*, and *-45* strongly responded to four abiotic stresses, suggesting that their encoded proteins play important roles in signaling and stress responses. To explore the potential functions of these proteins, we determined their location in the cell (such as the nucleus, cytoplasm, and cell membrane). We monitored the fluorescence from ZmbZIP17-green fluorescent protein (GFP), ZmbZIP33-GFP, ZmbZIP42-GFP, and ZmbZIP45-GFP fusion constructs as well as that of GFP driven by the CaMV35S promoter. When these fusion constructs were introduced into tobacco epidermal cells, the GFP-CaMV35S signal was consistently observed throughout the whole cell, whereas ZmbZIP17-GFP, ZmbZIP33-GFP, ZmbZIP42-GFP, and ZmbZIP45-GFP fusion proteins were restricted to the nucleus, as confirmed by 4′,6-diamidino-2-phenylindole (DAPI) staining. Thus, ZmbZIP17-GFP, ZmbZIP33-GFP, ZmbZIP42-GFP, and ZmbZIP45-GFP were confirmed to be nuclear proteins.

## 3. Discussion

Maize is an important staple food for people, a source of industrial raw materials, and the “king of feed” for livestock, and thus, it is an important crop for the livelihood of many people. However, maize is sensitive to high temperature, salt, and drought, and its production seriously declines under these stress conditions. The *bZIP* TFs play important roles in physiological processes, such as growth, development, and abiotic stress responses, but few studies have comprehensively analyzed *bZIP* families in maize. In this study, we conducted drought and rewatering transcriptomes analyses to identify differentially expressed *bZIP* genes between drought stress and rewatering in maize, and then comprehensively analyzed the 54 identified genes. The 54 genes formed nine groups in the composite phylogenetic tree (Figure 1). Gene classification and annotation analyses (Table S3) indicated that the functions of the genes within each group were similar, but there were also some differences. The genes in group A contained G-box-binding factor 4 and ABSCISIC ACID-INSENSITIVE 5-like proteins. It has been reported that group A *bZIPs* play important roles in stress- and ABA-response signaling networks in seeds and other plant tissues [4]. Group C genes encoded proteins with a basic leucine zipper motif and potential phosphorylation target sites for protein modification [45]. The other groups (D, G, H, I and S) contained genes encoding other TFs, such as TGA, HBP-1a, and G-box-binding factor 1, HY5, PosF21, and Ocs element-binding factor 1, respectively. This classification can be further rationalized by gene structures, additional conserved motifs, and cis-element analyses. 

To analyze the structure of the maize *ZmbZIP* genes, we conducted an exon/intron analysis. The number and arrangement of introns and exons provide information about gene family evolution and shed light on the origin and evolution of a given gene [46]. Besides the bZIP domain, several other conserved motifs have been detected in *bZIPs* of *Arabidopsis* [4], rice [5], and peach [47]. Gene structure and additional conserved motifs analyses indicated that differences in gene structure among different groups might be related to the functional diversity of maize *bZIP* members (Figure 2, Figure 3). In the same group, most maize *bZIPs* shared similar organization, including exon/intron distribution and motif components, but there were some differences. Most of the paralogous pairs had the same exons and motifs (such as *ZmbZIP12* and 31, *ZmbZIP39* and 45, *ZmbZIP23* and 7, and *ZmbZIP49* and 42). However, some paralogous pairs had different numbers of exons (7 and 4 exons in *ZmbZIP25* and *ZmbZIP18*, respectively; 8 and 13 exons in *ZmbZIP6* and *ZmbZIP32*, respectively) but had the same motifs. This may be due to the loss or gain of exons during gene evolution. In summary, each group of maize *bZIP* genes showed evolutionary conservation, but showed variations in genetic organization to some degree, suggesting that some maize *bZIP* members functionally diversified through differential amplification. In general, the results of structure and motifs analyses of the maize *bZIP* genes were consistent with the phylogenetic analysis, demonstrating the reliability of phylogenetic analysis, as well as the conservative evolutionary relationships among ZmbZIPs.

Gene duplication is one of the main drivers of genomic and genetic evolution and has been shown to play a key role in the expansion of many gene families [48], such as Homeodomain Leu zipper(HD-Zip) andHeat Shock Transcription Factor (HSF) families in maize [40,49]. In this study, we identified all the *bZIP* genes in maize. These genes clustered into nine groups: Some have evolved into larger groups, such as groups A, I, and S, while some groups have shown limited expansion, such as groups B, F, and H (Figure 1). Similar results have been observed for the *bZIP* genes in the genomes of *Arabidopsis*, rice, and sorghum [4,14,41], suggesting that the *bZIP* gene families in these plants might have expanded via common mechanisms. We found 18 gene pairs among the 54 maize *bZIP* genes (Figure 4), accounting for approximately 66.7% of the total *bZIP* genes (36/54). This result implied that gene duplication has contributed to the expansion of the maize bZIP family. Segmental duplication usually occurs in a slowly evolving gene family, such as the MYB gene family [50]. Therefore, we hypothesize that the maize *bZIP* gene family is a slowly evolving gene family, and that the segment duplications have played a key role in its expansion. It is more difficult for a gene family to expand via single-gene replication events. Thus, genomic replication is of great significance in expanding the repertoire of regulatory genes [51].

The *bZIP* TFs have been detected in most types of organisms. Among some Archaea species, only one member can be detected with the bZIP domain PF00170. The common ancestor in eukaryotes and prokaryotes, therefore, may be a single *bZIP* gene. Deppmann found that the original eukaryote *Giardia lamblia* also has only one *bZIP* gene in its genome, suggesting that a single *bZIP* gene is the common ancestor in eukaryotes [52]. However, the genome of the moss *Physcomitrella patens* encodes up to 40 *bZIP* TFs [14], suggesting that the *bZIP* family expanded after the divergence of this species from green algae. Our analyses showed that nine *Arabidopsis bZIP* genes had 16 orthologs in the maize genome. A similar result was observed when we aligned the Arabidopsis *bZIP* genes to the sorghum genome. However, 46 maize *bZIP* genes had 40 orthologs in sorghum (Figure 5). These results indicated that ZmbZIPs were more closely allied with SbbZIPs than AtbZIPs and that the large-scale expansion of these families may not have occurred before the monocot-dicot split.

With the development of high-throughput sequencing, PCA and gene co-expression network analyses have become important tools to screen genes closely related to target traits. A PCA can simplify the complex problem by replacing a larger number of original variables with fewer synthetic variables while minimizing the loss of data information. We found that *ZmbZIP22*, *-18*, *-54*, *-29* and, *-17* genes had higher absolute loadings on PC1. These genes may play important roles in the drought stress. Gene co-expression network analysis can reveal possible interactions among gene products by detecting similarities in gene expression. This can clarify intergenic interactions and identify core genes. In other studies, selected core genes have provided the molecular basis for the early diagnosis of fulminant hepatocellular carcinoma [53]. Key genes related to drought resistance have also been detected by gene co-expression analysis, and these data are valuable resources for further research on the molecular mechanisms of the drought stress response of *Cynanchum* and for the identification of drought-resistant genes [54]. In the present study, we identified some core bZIP genes, such as *ZmbZIP22*, *-18*, *-33*, *-4*, *-35*, *-5*, *-20*, *-17*, *-54*, and *-29*, through PCA and co-expression network map analyses. The products of these genes were predicted to be involved in protein transport, transcriptional regulation, and cell metabolism. It will be interesting to determine whether these genes are also core genes in the responses to other stresses. These results also may provide the molecular basis for drought-resistant breeding.

As noted in the introduction, plant *bZIP* TFs play vital roles in many biological processes, including growth, development, and responses to various abiotic and biotic stresses/signals [11]. To explore the tissue-specific expression patterns of *ZmbZIP* genes, the transcriptome data were analyzed to identify which genes were expressed in different organs and tissues (Supplemental Figure S2). The results indicated that certain *bZIP* genes may be expressed in a specific environment or at specific developmental stages. Some segmentally repeated *ZmbZIP* pairs also showed different expression profiles (such as *ZmbZIP12* and *ZmbZIP31*, and *ZmbZIP18* and *ZmbZIP25*), suggesting that functional diversification of duplicated genes has been a major feature of long-term evolution [55]. 

The *bZIP* TFs are involved in many biological processes and play an important role in the regulation of resistance to plant diseases, drought, and salt. In this study, we detected the expression patterns of different groups of *ZmbZIP* genes under four abiotic stresses. Many *bZIP* genes were up-regulated under PEG, ABA, NaCl, and high temperature stress (Figure 10). The highest transcript levels of *ZmbZIP17*, -*18*, *-33*, and *-42* under PEG stress were 85-times, 3-times, 8-times, and 61-times that in the control, respectively; under heat stress, their transcript levels were 6.8-times, 5.8-times, 2.5-times, and 12-times that in the control, respectively. In other words, the expression patterns of these genes were relatively consistent under PEG and heat stresses. This has also been observed for certain members of the *bZIP* family in *Arabidopsis*, apple, and grape [4,56,57]. Among the segmentally duplicated genes, most exhibited similar expression profiles under certain stress treatments, such as *ZmbZIP18*/*25* in response to all four stresses, *ZmbZIP20*/*21* in response to ABA and NaCl treatment, and *ZmbZIP39*/*45* in response to PEG and 37 °C treatment (Figure 10). This indicated that some duplicated genes might have redundant functions in response to specific stresses. However, some duplicated genes showed different expression patterns, indicative of new functions or sub-functionalization of the repeated genes, which are the main features of most repetitive genes [55,58].

**Figure 11:**
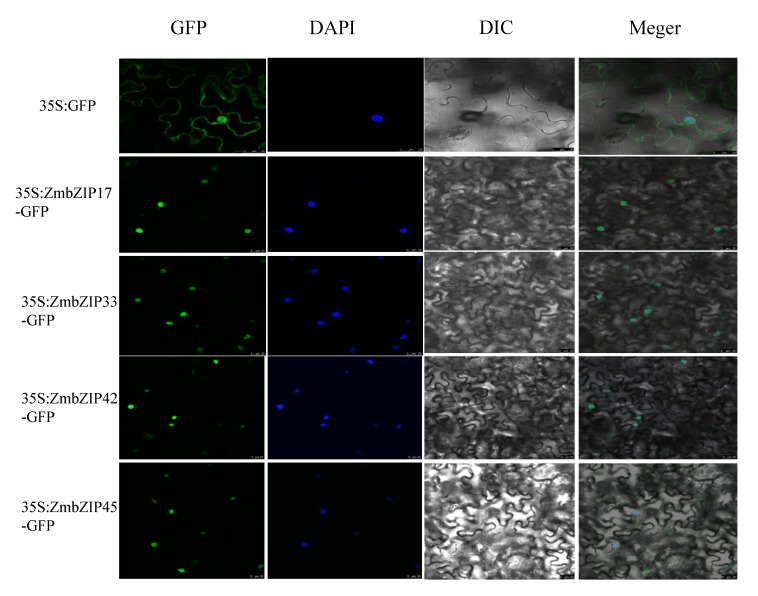
Subcellular localization of ZmbZIP17-GFP, ZmbZIP33-GFP, ZmbZIP42-GFP, and ZmbZIP45-GFP fusion protein. Fusion proteins were transiently expressed under control of the CaMV35S promoter in tobacco leaves and observed under a laser scanning confocal microscope. The green color is the green fluorescent protein (GFP) signal, and the blue color represents 4′,6-diamidino-2-phenylindole (DAPI), stained for the nucleus. Differential interference difference (DIC). Scale Bars = 25 μm.

We found that *ZmbZIP17*, *-33*, -*42*, and *-45* were highly induced under four stress treatments. Subcellular localization analyses confirmed that these genes encode nuclear proteins (Figure 11). Based on these results, we hypothesized that *ZmbZIP17*, *-33*, *-42*, and *-45* may have similar functions in response to abiotic stress, although these functions are yet to be confirmed experimentally. It has been reported that members of the A subfamily play major roles in the responses to stress, such as ABA stress [59]. Members of the D group of *bZIPs* are involved in disease resistance and developmental regulation [59], while members of the S subfamily are involved in sucrose signaling and regulation of the stress response [60]. The results of our study are consistent with those reported for other plants, but there are also differences. For example, in maize, members of the A subfamily are mainly involved in the ABA response, while members of groups C, D, and S are induced by ABA and other stresses, but to different degrees. Our results indicate that the *bZIP* family members and groups coordinate to tightly regulate growth, development, and responses to abiotic stresses.

## 4. Materials and Methods

### 4.1. Plant Materials and Stress Treatment

Full-fledged inbred line of Yu 882 seeds were selected and rinsed with 2% H_2_O_2_ for 10 min. The seedlings were grown in a greenhouse at a light/dark light cycle of 14 h/10 h, 60% relative humidity, and a light intensity of 120 μmol m^−2^ s^−1^. Seedlings were grown in Hoagland’s nutrient solution (pH 5.8), which was refreshed every 2 day. The leaves of the three-leaf stage were treated with 20% polyethylene glycol (add 20% PEG to Hoagland’s nutrient solution), treated for 60 h and 96 h, and rewatered for 3 d, denoted as T60, T96, and T3d, and the control groups (Hoagland’s nutrient solution) were named CK60, CK96, and CK3d, respectively. These samples were sequenced by transcriptome. At the same time, seedlings at the 3-fully expanded leaf stage were transferred to the nutrient solution containing 20% PEG 6000, NaCl (200 mmol L^−1^), ABA (5 μmol L^−1^), and 37 °C, respectively. After 0 h, 4 h, 8 h, 12 h, and 24 h, the leaves of maize were collected and immediately stored at −80 °C. Three plants from three different containers of each treatment were used as biological replicates.

### 4.2. RNA Isolation and qRT-PCR Analysis

A total of 15 *bZIP* genes screened for all analyses were quantified by quantitative real-time PCR (qRT-PCR). We extracted RNA from the leaves of three independent biological replicates for each at 0 h, 4 h, 8 h, 12 h, and 24 h. First-strand cDNA was synthesized using a Hifair^®^ II 1st Strand cDNA Synthesis SuperMix (YEASEN, Shanghai, China). Gene-specific primers for qPCR were designed based on the corresponding sequence using Primer5 and are listed in Table S4. Actin 18s was used as an internal control. The qPCR analyses were carried out using Hieff^®^ qPCR SYBR^®^ Green Master Mix (YEASEN, Shanghai, China) on a Light Cycler 480 instrument (Roche, Basel, Switzerland), according to the manufacturer’s instructions. Three technical replicates were analyzed for each gene. The relative expression level (2^−∆∆Ct0 h^) in the control plants without treatment was normalized to 1.

### 4.3. Collection and Classification of bZIP Transcription Factors

The resulting reads were aligned to the *Z. mays* genome that was retrieved from NCBI. The transcriptome data have been deposited to the sequence read archive (SRA) under the accession number PRJNA477643. We identified genes with |log2 (fold change)| ≥ 2 and a false discovery rate (FDR) < 0.05 in a comparison as significant differentially expressed genes (DEGs). Using Pfam and SMART programs to screen for DEGs with bZIP domains, 54 significant differentially expressed ZmbZIPs were then screened. Phytozome was used to download the amino acid sequences and chromosome positions of these genes as well as the homologous Arabidopsis and sorghum. The molecular weight (MW) and isoelectric point (PI) were predicted by ProtParam (http://web.expasy.org/protparam/). Meanwhile, the DEGs were then subjected to enrichment analysis of GO functions and expression cluster analysis.

### 4.4. Compound Phylogenetic Tree, Gene Structure, Additional Conserved Motifs Analysis, and Chromosomal Locations

Full-length protein sequence alignments of *ZmbZIPs* and the homologous *Arabidopsis* and sorghum family were generated using the MEGA (https://www.megasoftware.net)and then manually adjusted to the alignment. The phylogenetic tree was constructed in MEGA6 using neighbor-joining (NJ). The DNA and cDNA sequences of each *ZmbZIPs* were downloaded from the MaizeGDB (https://www.maizegdb.org/), and the gene structure of exon-intron were analyzed online using the Gene Structure Display Server 2.0 (http://gsds.cbi.pku.edu.cn/). We used the online software Multiple Expectation maximization for Motif Elicitation (MEME) program (http://meme-suite.org/tools/meme) to query the motifs of maize bZIP proteins (Table S5). The Chromosomal localizations information of *ZmbZIPs* were received from the gramene database (http://ensembl.gramene.org/genome_browser/index.html). The distribution of *ZmbZIP* genes on the maize chromosomes was drawn by MapInspect (http://mapinspect.software.informer.com/) and modified manually with annotation.

### 4.5. Interspecies Microsynteny Analysis

In order to detect the homogenous region between maize, sorghum, and *Arabidopsis*, a multiple sequence alignment was used to detect the protein sequences of maize, sorghum, and *Arabidopsis*, with the similarity of >70%. Subsequently, the MCScanX (http://chibba.pgml.uga.edu/mcscan2/) and associated downstream tools using default parameters were used for detecting the collinear blocks. Finally, the relationships of *bZIPs* orthologous genes among the three species were plotted using Circos software (http://circos.ca/).

### 4.6. Cis-Elements in the Promoter Regions of Abiotic Stress-Responsive and Microarray-Based Expression Analysis of ZmbZIP Genes

To predict cis-acting regulatory DNA elements (cis-elements) in promoter regions of maize *bZIP* genes, the PLACE website (Available online: http://www.dna.affrc.go.jp/PLACE/signalscan.html) was adopted to identify putative cis-elements in the 2000 bp genomic DNA sequences upstream of the start codon (ATG).

We obtained transcriptome data from the maize genome mapping article developed by RNA sequencing and comparative evaluation of transcriptomes based on RNA sequencing and microarrays. The average gene expression value must be greater than 0 Fragments Per Kilobase Exon model per Million mapped fragments (FPKM) in at least one of the 10 tissues.

### 5.7. The PCA and Co-Expression Network Map Analysis of ZmbZIP Genes

The correlation coefficient between genes was calculated based on the expression levels (FPKM) of 54 *ZmbZIP* genes in 18 samples (CK60-1, CK60-2, CK60-3, CK96-1, CK96-2, CK96-3, CKR3d-1, CKR3d-2, CKR3d-3, T60-1, T60-2, T60-3, T96-1, T96-2, T96-3, T3d-1, T3d-2, T3d-3), and then the internal steps of the R package (version 3.5.3) were used for principal component analysis (PCA). We imported the filtered network into Cytoscape to create a co-expression network map.

### 5.8. Determination of Subcellular Localization of ZmbZIPs

A complete open reading frame (ORF) of *ZmbZIP17*, *ZmbZIP33*, *ZmbZIP42*, and *ZmbZIP45* was PCR-amplified and the primers are shown in Table S4. These four cDNA sequences were cloned between SpeI and BamHI sites (underlined in primer sequences) of the pMDC83-GFP vector. The resulting 35S: ZmbZIP17-GFP, 35S: ZmbZIP33-GFP, 35S: ZmbZIP42-GFP, 35S: ZmbZIP45-GFP, and GFP control vector were transiently expressed in *Nicotiana benthamiana* leaves via Agrobacterium-mediated infiltration [61]. Two days later, the fluorescence of the infected leaf tissue was observed under a Zeiss LSM700 (Zeiss, Jena, Germany) confocal microscope and the DNA dye 4,6-diamidino-2-phenylindole (DAPI) was used to visualize the nucleus.

## 5. Conclusions

We analyzed the phylogenetic relationships and gene structure of 54 differentially expressed *ZmbZIP* genes between the maize drought and rewatering transcriptomes. The results indicated that the conservation of the *bZIP* gene family during evolution has been accompanied by differentiation to some extent. The bZIP gene family has expanded during evolution as a result of small-scale repetitive events (such as segmental duplication). The PCA and gene co-expression analyses identified 10 core *bZIP* genes. Analyses of their transcript levels under four abiotic stresses indicated that *ZmbZIP17*, *-33*, *-42*, and *-45* actively participate in abiotic stress responses and their proteins are localized in the nucleus. From an applications point of view, the results of this study provide a useful reference for more detailed *bZIP* functional analyses and help us to understand how *bZIP* transcription factors positively and negatively regulate gene expression. These data also represent an excellent molecular resource for drought-resistance breeding, for molecular marker-assisted selection, and for the generation of new corn varieties with stronger resistance to biotic and abiotic stresses.

## Figures and Tables

**Table 1:** The detailed information of maize basic leucine zipper (*bZIP*) family members.

*Gene Name*	ID Gene Identifier	Gene Names Description	Protein Size (aa)	Chromsome	5′ End	3′ End	Molecular Weight (kDa)	Theoretical pI	Instability Index	GRAVY
*ZmbZIP1*	GRMZM2G000171	Basic leucine zipper 19	332	Chr5	61724734	61728541	35.43	6.16	44.93	−0.34
*ZmbZIP2*	GRMZM2G000842	TGACG motif-binding transcription factor 6	542	Chr6	149721944	149728050	60.34	8.6	61.64	−0.451
*ZmbZIP3*	GRMZM2G007063	Basic leucine zipper 25	410	Chr5	4253958	4257719	42.88	5.23	55.72	−0.467
*ZmbZIP4*	GRMZM2G008166	ABA-INSENSITIVE 5	194	Chr6	111882466	111885951	21.58	9.85	31.95	−0.303
*ZmbZIP5*	GRMZM2G011932	G-box binding factor 1	377	Chr6	165543429	165548373	40.12	7.11	64.81	−0.678
*ZmbZIP6*	GRMZM2G019106	Transcription factor HBP-1a	361	Chr4	239534487	239538429	38.21	5.31	58.4	−1.017
*ZmbZIP7*	GRMZM2G020799	Basic leucine-zipper 58	176	Chr5	204603643	204604321	20.27	6.52	78.7	−0.882
*ZmbZIP8*	GRMZM2G024851	DNA binding protein	348	Chr3	138906763	138910797	39.14	8.8	70.2	−0.966
*ZmbZIP9*	GRMZM2G045236	DNA binding protein	654	Chr6	147087480	147092015	69.66	9.03	43.59	−0.49
*ZmbZIP10*	GRMZM2G050912	Putative *bZIP* transcription factor	272	Chr9	93665910	93671589	28.53	8.94	74.35	−0.616
*ZmbZIP11*	GRMZM2G060109	Putative *bZIP* transcription factor	563	Chr2	214613200	214616521	59.82	5.48	53.55	−0.37
*ZmbZIP12*	GRMZM2G062391	Basic leucine-zipper 52	346	Chr1	56217970	56223418	37.38	6.43	50.64	−0.635
*ZmbZIP13*	GRMZM2G066734	*bZIP* transcription factor 53	129	Chr9	135645489	135646430	14.48	10.89	68.73	−0.417
*ZmbZIP14*	GRMZM2G073427	Basic leucine zipper 25	310	Chr1	170592733	170597621	33	5.19	45.97	−0.525
*ZmbZIP15*	GRMZM2G073892	*bZIP* transcription factor	171	Chr5	217591763	217592969	18.62	9.22	83.06	−0.743
*ZmbZIP16*	GRMZM2G092137	*bZIP* transcription factor	156	Chr7	83788712	83791125	17.57	9.24	82.49	−0.835
*ZmbZIP17*	GRMZM2G093020	common plant regulatory factor	106	Chr1	143352528	143354301	11.89	10.7	67.32	−1.169
*ZmbZIP18*	GRMZM2G094352	Transcription factor TGA4	384	Chr10	49989443	49992484	42.53	8.9	56.89	−0.376
*ZmbZIP19*	GRMZM2G095078	EM binding protein 1-like protein	386	Chr7	19265565	19270520	40.28	6.28	69.32	−0.69
*ZmbZIP20*	GRMZM2G098904	Basic leucine zipper 9	324	Chr5	87150348	87153356	34.29	5.37	57.99	−0.373
*ZmbZIP21*	GRMZM2G103647	Basic leucine zipper 9	282	Chr9	109162612	109164995	30.14	5.2	54.59	−0.526
*ZmbZIP22*	GRMZM2G120167	DNA binding protein	349	Chr4	16152931	16158076	38.56	9.49	66.14	−0.936
*ZmbZIP23*	GRMZM2G122846	Putative *bZIP* transcription factor	150	Chr4	165667701	165668420	16.79	9.6	71.41	−0.551
*ZmbZIP24*	GRMZM2G129247	G-box-binding factor 4	254	Chr6	150274418	150276924	27.09	7.76	68.69	−0.585
*ZmbZIP25*	GRMZM2G131961	TGACG motif-binding transcription factor 4	405	Chr2	6719168	6724854	45.34	7.18	53.72	−0.394
*ZmbZIP26*	GRMZM2G132868	G-box-binding factor 4	257	Chr8	100221799	100225360	27.04	6.79	66.88	−0.532
*ZmbZIP27*	GRMZM2G136266	Transcription factor PosF21	398	Chr10	7921801	7925637	42.99	6.72	60.01	−0.813
*ZmbZIP28*	GRMZM2G137046	Transcription factor HY5	170	Chr5	112670143	112675317	18.72	9.89	58.5	−1.118
*ZmbZIP29*	GRMZM2G138340	ABRE-binding factor Embp-2	140	Chr1	291173937	291177741	14.85	4.77	51.14	−0.714
*ZmbZIP30*	GRMZM2G146020	Transcription factor PosF21	321	Chr3	142765244	142769768	34.63	6.42	62.81	−0.698
*ZmbZIP31*	GRMZM2G151295	Basic leucine-zipper 52	353	Chr9	131353502	131359160	38.05	6.14	55.83	−0.645
*ZmbZIP32*	GRMZM2G153144	Transcription factor HBP-1a	350	Chr5	74180653	74185259	37.32	5.42	57.41	−1.019
*ZmbZIP33*	GRMZM2G157722	ABA-INSENSITIVE 5	356	Chr4	57698284	57703555	37.69	5.44	49.79	−0.379
*ZmbZIP34*	GRMZM2G159134	ABA-INSENSITIVE 5	333	Chr3	186805980	186810898	36.25	6.69	59.57	−0.71
*ZmbZIP35*	GRMZM2G160136	putative *bZIP* transcription factor	251	Chr3	47233315	47236145	27.59	9.36	60.85	−0.369
*ZmbZIP36*	GRMZM2G160902	*bZIP* transcription factor 53	152	Chr3	128957708	128959418	16.71	9.09	78.39	−0.611
*ZmbZIP37*	GRMZM2G161009	ABA-INSENSITIVE 5	324	Chr8	119246143	119249647	35.33	6.38	52.91	−0.657
*ZmbZIP38*	GRMZM2G171370	G-box-binding factor 1	435	Chr2	161590575	161596682	45.47	6.75	63.95	−0.809
*ZmbZIP39*	GRMZM2G174284	TGACG motif-binding transcription factor 6	335	Chr1	51753616	51759590	37.43	8.59	56.99	−0.524
*ZmbZIP40*	GRMZM2G175280	DNA binding protein	465	Chr1	197089134	197092935	49.71	7.15	73.46	−0.736
*ZmbZIP41*	GRMZM2G175870	DNA binding protein	229	Chr6	156230705	156237177	24.84	5.36	48.29	−0.49
*ZmbZIP42*	GRMZM2G177046	Ocs element-binding factor 1	185	Chr1	281401644	281402596	20.19	10.5	71.82	−0.443
*ZmbZIP43*	GRMZM2G180847	Putative transcription factor PosF21	371	Chr2	194071950	194077869	39.35	5.96	59.58	−0.655
*ZmbZIP44*	GRMZM2G361611	Ocs element-binding factor 1	162	Chr4	239887667	239890292	17.13	6.29	52.56	−0.107
*ZmbZIP45*	GRMZM2G361847	TGACG motif-binding transcription factor 6	333	Chr7	174267403	174273062	37.15	8.9	55.08	−0.521
*ZmbZIP46*	GRMZM2G368491	Common plant regulatory factor 7	142	Chr3	227566001	227567569	15.53	9.75	50.25	−0.523
*ZmbZIP47*	GRMZM2G425920	Transcription factor HY5-like	215	Chr10	122253987	122259270	23.6	10.38	69.63	−1.171
*ZmbZIP48*	GRMZM2G438293	ABA-INSENSITIVE 5	238	Chr8	165990148	165991775	24.92	5.73	68.24	−0.524
*ZmbZIP49*	GRMZM2G438652	Basic leucine zipper 25-like	214	Chr7	3077372	3078435	22.3	11.35	59.78	−0.129
*ZmbZIP50*	GRMZM2G448607	Ocs element-binding factor 1	135	Chr6	24280540	24281904	15.01	10.14	71.91	−0.733
*ZmbZIP51*	GRMZM2G043600	Transcription factor RF2a-like	275	Chr2	216709894	216711451	30.53	6.88	66.56	−0.907
*ZmbZIP52*	GRMZM2G479760	ABA-INSENSITIVE 5	377	Chr5	210099032	210100747	40.16	10.03	57.18	−0.659
*ZmbZIP53*	GRMZM2G479885	Ocs element-binding factor 1	151	Chr1	147170752	147172309	16.86	6.93	73.52	−0.716
*ZmbZIP54*	GRMZM5G858197	G-box-binding factor 4	260	Chr3	175672623	175675787	27.55	8.95	74.98	−0.673

## Supplementary Materials

Supplementary materials can be found at https://www.mdpi.com/1422-0067/20/17/4103/s1.
