# Supplementary material for: Systematic Analysis of Differentially Expressed Maize ZmbZIP Genes between Drought and Rewatering Transcriptome Reveals bZIP Family Members Involved in Abiotic Stress Responses
Source: Int J Mol Sci. 2019 Aug 22;20(17):4103. doi: 10.3390/ijms20174103 (PMC6747360; doi:10.3390/ijms20174103)
Supplement: Supplementary file 1 [file ijms-20-04103-s001.zip › ijms-557587-supplementary/Supplemental/Supplemental Figures.doc]

Supplemental Figure S1. Cis-elements in the promoter regions of maize bZIP genes. Different colors represent different groups (from top to bottom are groups A, B, C, D, F, G, H, I, S).The stress-responsive cis-elements are distributed on the grey line. The ABRE, ARE, STRE, DRE core and MBS sequences are respectively represented by rectangles of different colors. ABRE: ABA responsive element; ARE: antioxidant responsive element; STRE: heat responsive element; DRE core and MBS: dehydration-responsive element.

Supplemental Figure S2. Expression profiles of maize ZmbZIP genes across different tissues. Clustering according to groups, different colors represent different groups (from top to bottom are groups A, B, C, D, F, G, H, I, S) .Genes highly or weakly expressed in the tissues is shown in red and blue, respectively.

Supplemental Figure S3.The proportion of each Principal component of the *ZmbZIP* gene.Different colors express different ZmbZIP genes, and genes with close colors indicate that the expression trends of these genes are more consistent.
